# Pre-return to work consultation and therapeutic part-time work: Cross-sectional study on level of knowledge and use by general practitioners in France

**DOI:** 10.1080/13814788.2021.1948007

**Published:** 2021-07-14

**Authors:** Cyril Bègue, Lucille Desmidt, William Bellanger, Christine Tessier-Cazeneuve, Audrey Petit, Anne-Laure Couturier

**Affiliations:** a Univ Angers, Département de Médecine Générale, Angers, France; b Univ Angers, Univ Rennes, Inserm, EHESP, Irset (Institut de recherche en santé, environnement et travail) - UMR_S 1085, SFR ICAT, Angers, France; c Univ Angers, CHU Angers, Univ Rennes, Inserm, EHESP, Irset (Institut de recherche en santé, environnement et travail) - UMR_S 1085, SFR ICAT, Angers, France

**Keywords:** General practitioners, return to work, tool use behaviour, occupational health physicians

## Abstract

**Background:**

In France, general practitioners (GPs) may use two tools specifically designed to help employees who experience difficulties in returning to work after sick leave: the pre-return-to-work (PRW) medical consultation and therapeutic part-time (TPT) work.

**Objectives:**

The objective was to investigate the level of knowledge and use of these two tools by GPs in Maine-et-Loire, France.

**Methods:**

This cross-sectional study was performed using a telephone questionnaire to evaluate the level of knowledge of GPs and the use of these two tools in patients having difficulties returning to work.

**Results:**

Among the 200 randomly selected GPs, 122 responded (response rate: 61%). More than half of the interviewed GPs declared they ‘often’ (46%) or ‘always’ (14%) contacted the occupational physician in these situations. Moreover, 62.2% and 32.7% believed that they had a ‘vague’ or ‘very good’ level of knowledge, and 41% and 51% declared either ‘frequent’ or ‘regular’ level of use of the PRW medical consultation, respectively. Regarding TPT work, 47% and 53% reported a ‘very good’ or ‘vague’ level of knowledge, and 41% and 51% a ‘frequent’ or ‘regular’ level of use, respectively. GPs who had a better level of knowledge of this tool reported a higher level of use (*p* < 0.001).

**Conclusion:**

This study shows that while the level of knowledge and use of the PRW medical consultation and TPT work is good, it is not optimal. This could be improved by organising training courses for GPs. Obstacles to their wider use could be investigated further in a qualitative study.

KEY MESSAGES

- Pre-return-to-work medical consultation and therapeutic part-time work appear to be underused by GPs.
- The level of knowledge regarding these tools is reasonable and associated with use.
- GPs do not systematically contact the OP in case of work-related problems.

## Introduction

The employment rate of people with a health problem or disability is just over half that of unaffected individuals [1]. Given the social, economic and psychological consequences of unemployment, there is an urgent need to address work-related health problems and sick leave [2]. Among health professionals, general practitioners are best placed to address work-related health problems. In fact, 83% of French people report visiting their general practitioner (GP) at least once a year and nearly three-quarters of periods of sick leave are prescribed by a GP [3,4].

When faced with patients with health problems that may compromise maintaining employment, GPs need to identify underlying difficulties. Besides managing the health problems, GPs also facilitate a return to work. In France, two return-to-work and stay-at-work tools are available: referral to the Occupational Physician (OP) for a ‘pre-return-to-work’ (PRW) medical consultation and prescription of ‘therapeutic part-time’ (TPT) work. These tools are defined by a legal framework and implemented in collaboration with the patient and the various parties involved. French health authorities recommend using pre-resumption visits as well as therapeutic part-time work to encourage maintenance of employment, even in the absence of studies attesting to the effectiveness of these measures [5].

A PRW consultation is required when sick leave exceeds three months and may be requested either by GPs, practitioners from the French national health insurance system or patients, but not employers. During a PRW consultation, the OP discusses options for remaining in employment with the patient and identifies potential difficulties and possible solutions. The OP can recommend work adjustments, suggest redeployment, training, or a career change. Several PRW consultations may occur during the same period of sick leave. During a PRW consultation, the OP clinically assesses whether the employee can return to work or not. However, the administrative decision by the OP regarding work resumption (‘aptitude’ to occupy that job) cannot be taken during the sick leave period of the employee. At the end of the PRW consultation, the OP informs the employer of the recommendations given to the employee, except if the employee refuses.

Where a progressive return to work might benefit the employee’s health, or if the employee requires retraining or occupational rehabilitation, the OP can suggest TPT work during the PRW consultation. The GP may also recommend and prescribe a progressive return to work. Before returning to work, the OP informs the employer about the recommended TPT work conditions (percentage of activity, duration, and work hours). Employers can refuse these recommendations but must justify their decision.

If difficulties occur returning to work, GPs should contact the OP directly (by letter, mail or telephone) or indirectly (e.g. letter for the OP given to the patient, or by suggesting the patient contact the OP) to discuss the patient’s situation. In France, each company must have a dedicated OP for employees, either within the company or as an inter-company service. GPs must therefore interact with various OPs.

In France, few studies have investigated how GPs encourage return to work after sick leave. Studies conducted in the South-East of France between 2005 and 2009 showed that GPs found it difficult to implement the available tools for remaining in employment [6–8]. A study published by the French institute for prevention and health education (INPES) in 2012 confirmed that GPs required more training in this field. Interviewed GPs indicated that they often proposed a PRW consultation, while only 8% never did [9]. However, a study published in 2016 showed that the PRW consultation is underused by GPs, with only 16% of PRWs referred by GPs [10].

The main objective of this study was to evaluate the level of knowledge concerning PRW consultations and TPT work, and their use by French GPs in the Maine-et-Loire department.

## Methods

### Selection of study subjects

**Figure 1.**
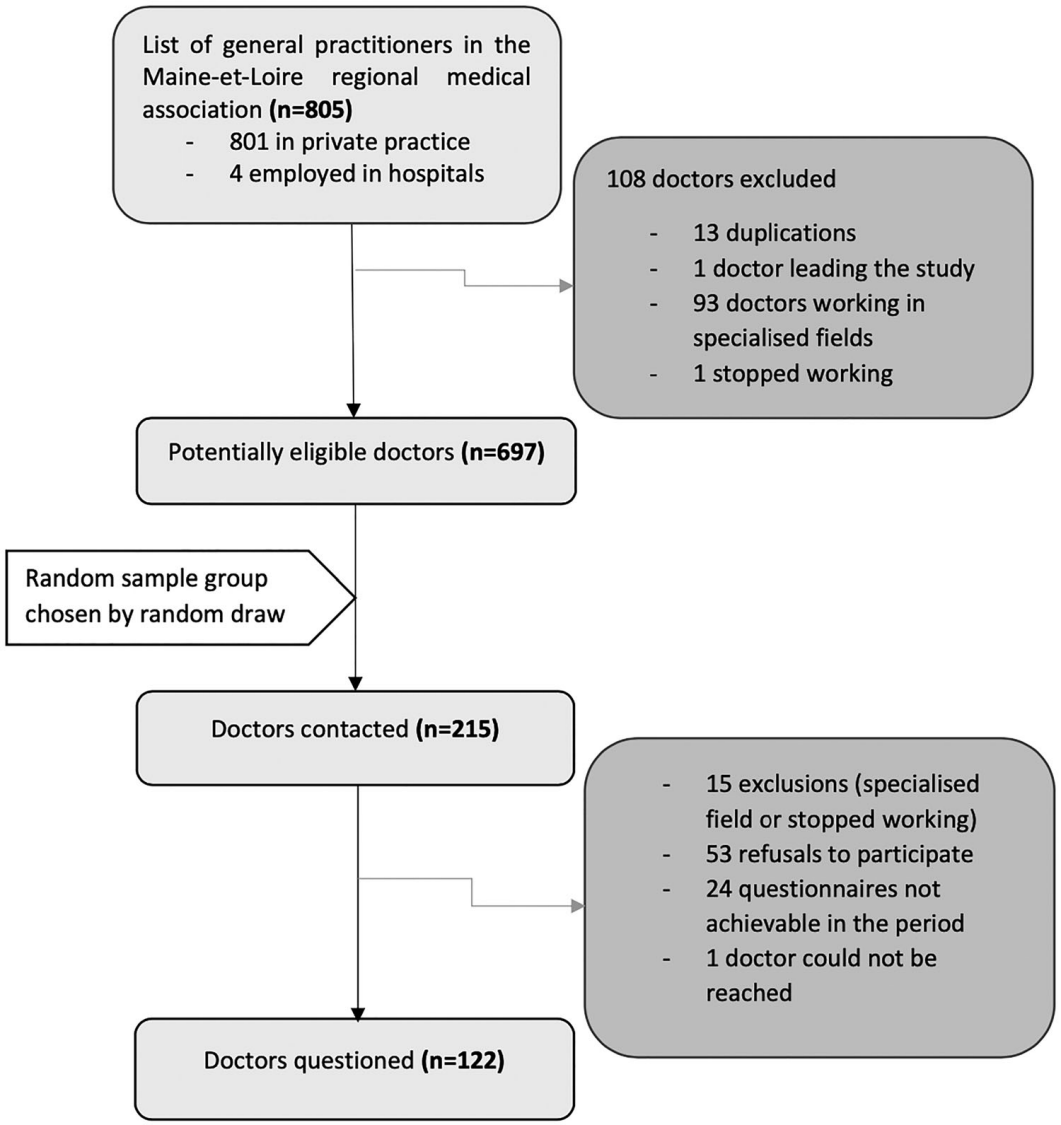
Study flowchart.

For this cross-sectional study, all GPs working in primary care were selected from the list of GPs registered in the Maine-et-Loire medical association. GPs who were specialised exclusively in a field that does not include sick leave prescription were excluded from the study. Of all 805 GPs listed in the association, 697 were eligible for the study and were classified in random order using Excel^®^ software (Figure 1). The objective was to obtain at least 100 responses to ensure feasibility and allow subgroup analyses. Assuming a 50% response rate, this meant contacting 200 eligible GPs.

### Study design

Selected GPs were contacted by telephone by two authors between late December 2016 and mid-January 2017 for harmonised interviews. Some information on the project (study objective, anonymity) was given verbally, and verbal non-opposition to the questionnaire was considered consent. Data was collected using a questionnaire developed based on published reports and expert opinion and was then tested. Questionnaires were completed in harmonised telephone interviews.

### Measurements

**Figure 2.**
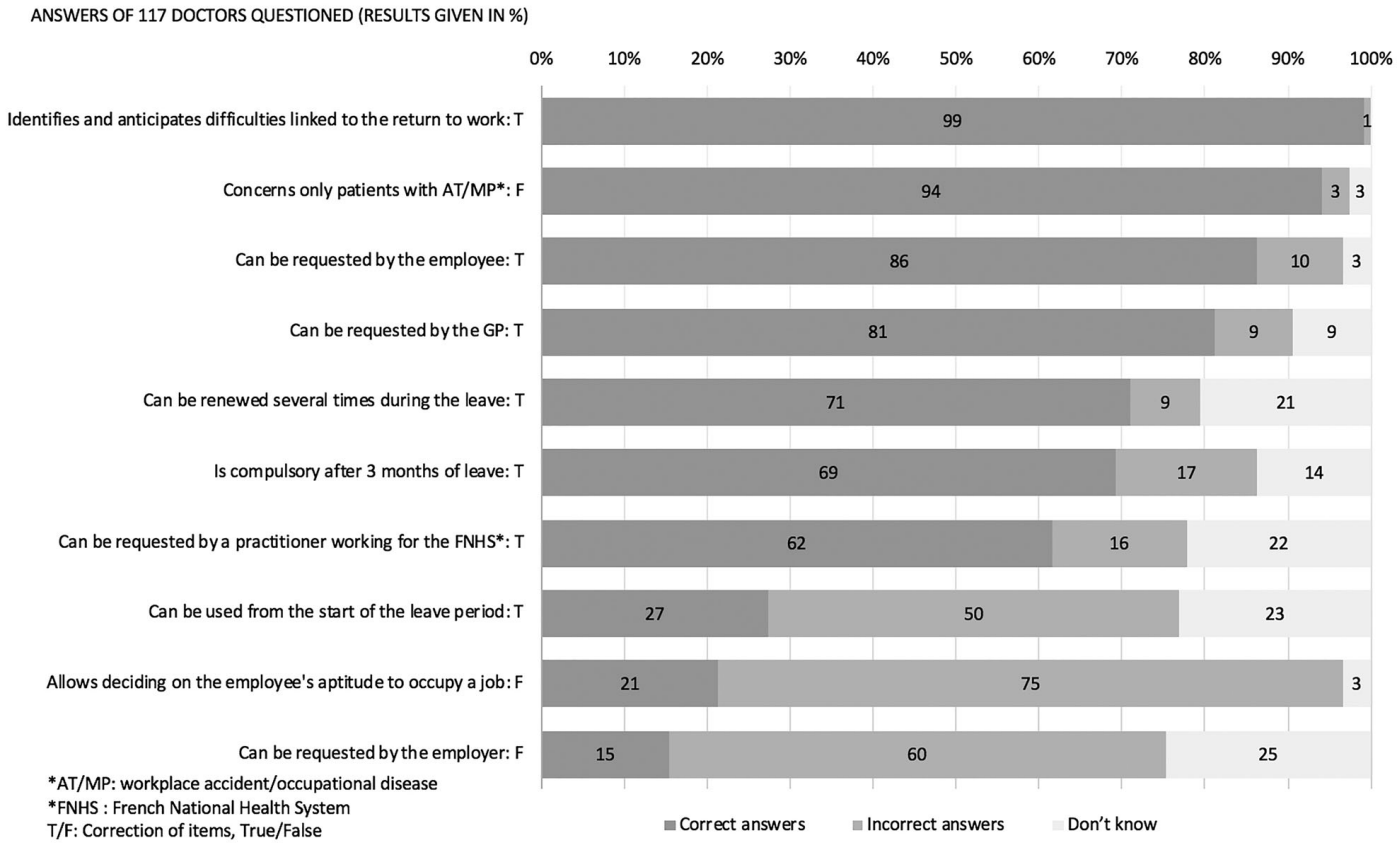
Distribution of answers to the questions concerning pre-return-to-work (PRW) consultation.

**Figure 3.**
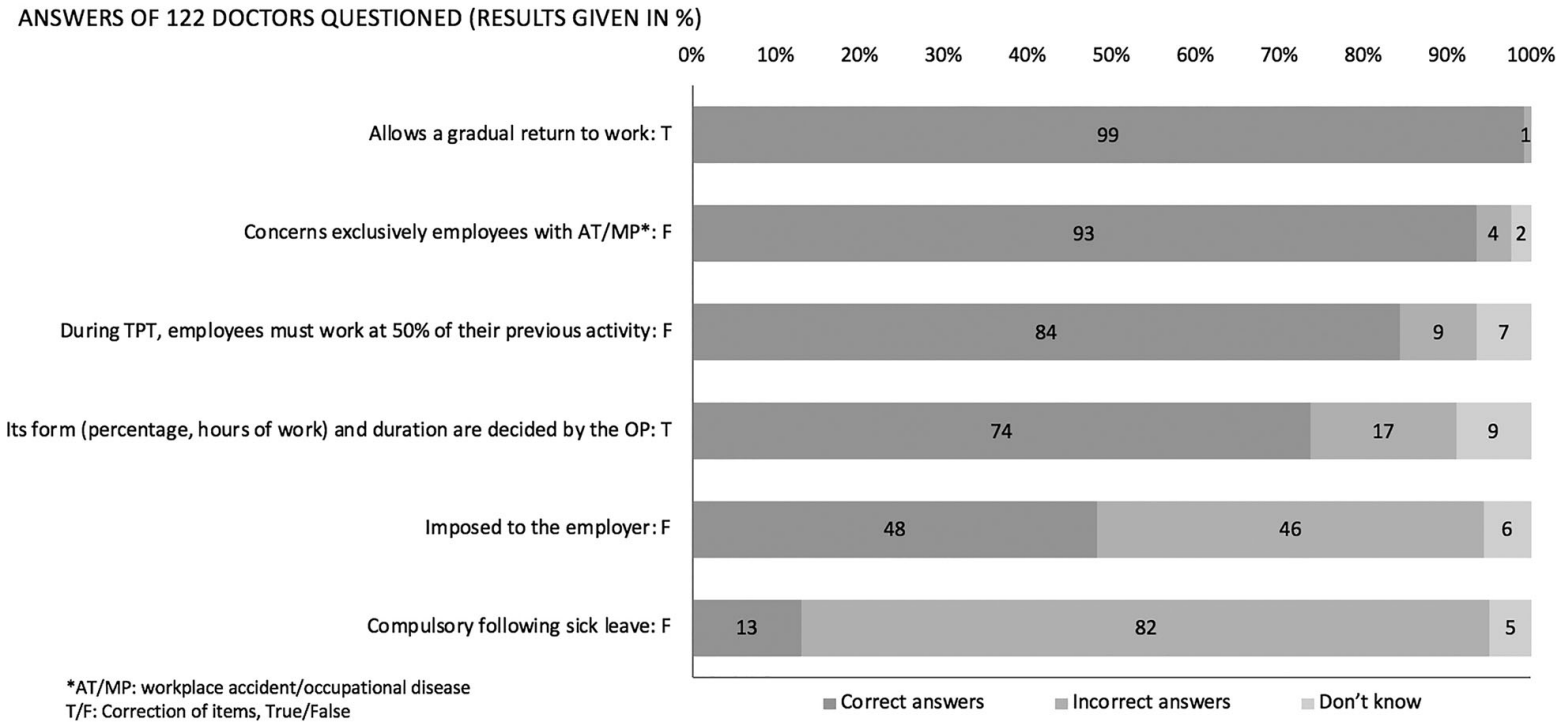
Distribution of answers to questions concerning therapeutic part-time (TPT) work.

The interviews were conducted in the following manner. First, GP socio-demographic data and practice type were collected. Then, the frequency by which GPs contacted the Ops and knowledge and frequency of use of each tool in complicated return to work situations were evaluated. These were measured using Likert scales with three responses for knowledge (not at all, vaguely, and very well) and four points for frequency (never, rarely, often, and always). For the analysis, these answers were transformed to ‘no’, ‘vague’ and ‘very good’ for levels of knowledge, and ‘no’, ‘rare’, ‘frequent’ and ‘regular’ for levels of use. Therefore, a ‘regular’ level of PRW consultation use indicates that GPs said they always recommend a PRW consultation to a patient during an extended period of sick leave. For GPs who said they knew the two tools ‘vaguely’ or ‘very well’, their knowledge was evaluated using closed-ended questions (see Figures 2 and 3 for the lists of questions). One point was given for each correct answer.

### Statistical analysis

Data were anonymised. The GP characteristics were described using mean, median and percentage values.

Statistical analyses of sub-groups were carried out using Chi-square or Fisher’s exact tests, for qualitative data, and Student’s *t*-test for quantitative data. A *p*-value <0.05 was considered significant.

### Ethics

The study was approved by Angers University Hospital ethics committee (2016/120) and was declared to the data protection officer (CIL) at Angers University, France.

## Results

In total, among the 215 GPs contacted, 200 were eligible and included, of which 122 completed the telephone questionnaire (response rate = 61%) (Figure 1). Among these 122, 66% were men and the mean age was 50 years. Most of the GPs (80%) worked in a group practice (Table 1). The gender and practice type distribution did not differ between responders and non-responders.

**Table 1.** Characteristics of the general practitioner (GP) sample.

	Interviewed GPs, *n* = 122
Sex	
Women	41 (34%)
Men	81 (66%)
Age	
25–34 years	13 (11%)
35–44 years	28 (23%)
45–54 years	31 (25%)
55–64 years	47 (39%)
≥65 years	3 (2%)
Practice type	
Individual practice	25 (20%)
Group of GPs	73 (60%)
Multidisciplinary health centre	24 (20%)

Sixty percent of the GPs declared that they ‘often’ (46%) or ‘always’ (14%) directly contacted the OP when the return to work may be complicated. Only 9% reported never contacting the OP, and 31% rarely contacted them.

Moreover, concerning the level of knowledge about the PRW consultation, most respondents (64%) rated their knowledge as vague and 32% very good. The percentage of the GPs who reported having a very good level of knowledge was higher among those 45 years or older (41% vs. 15%; *p* < 0.01) (Table 2).

**Table 2.** Level of knowledge concerning the pre-return-to-work (PRW) consultation and therapeutic part-time (TPT) work according to the general practitioner (GP) age group and practice type (total *n* = 122).

	No, *n* (%)	Vague	Very good	*p*-Value^†^
PRW				
<45 years of age	1 (2)	34 (83)	6 (15)	**
≥45 years of age	4 (5)	44 (54)	33 (41)	
Individual practice	1 (4)	17 (68)	7 (28)	NS
Group practice	4 (4)	61 (63)	32 (33)	
Total (*n* = 122)	5 (4)	78 (64)	39 (32)	
TPT				
<45 years of age	0	25 (61)	16 (39)	NS
≥45 years of age	0	40 (49)	41 (51)	
Individual practice	0	12 (48)	13 (52)	NS
Group practice	0	53 (55)	44 (45)	
Total (*n* = 122)	0	65 (53)	57 (47)	

^†^Fisher’s exact test.

**p < 0.01; NS: not significant.

Concerning the actual knowledge about the PRW consultation, 72% of the GPs responded correctly to at least six out of ten questions. 7 of 10 questions concerning PRW were correctly answered by most (62–99%) of the GPs (Figure 2). However, only 21% knew that the PRW consultation could not be used to decide whether or not a patient can perform a given occupation. Moreover, only 27% of GPs knew that the PRW consultation could be used from the start of the sick leave period, and 15% of GPs knew that the employer could not request the PRW consultation. No significant association was found between correct responses to these three questions and GP age or practice type. The total score was higher in the GP group with a very good level of knowledge than in the group with a vague level of knowledge (6.79/10 vs. 6/10; *p* < 0.01).

Among GPs with some knowledge about the PRW consultation (*n* = 117), 41% considered using the consultation frequently and 51% regularly (Table 3). Also, 97% of respondents with a very good level of knowledge concerning the PRW consultation also reported using the consultation frequently or regularly, compared with 90% of respondents who had only a vague level of knowledge (no significant difference).

**Table 3.** Level of frequency of use of pre-return-to-work (PRW) and therapeutic part-time (TPT) work according to the general practitioner (GP) age group and type of practice.

	Never, *n* (%)	Rare	Frequent	Regular	*p*-Value^†^
PRW					
<45 years of age	1 (3)	3 (8)	13 (33)	23 (58)	NS
≥45 years of age	0 (0)	5 (6)	35 (45)	37 (48)	
Individual practice	0 (0)	4 (17)	7 (29)	13 (54)	NS
Group practice	1 (1)	4 (4)	41 (44)	60 (51)	
Total (*n* = 117)	1 (1)	8 (7)	48 (41)	60 (51)	
TPT					
<45 years of age	1 (2)	6 (15)	34 (83)	0 (0)	*
≥45 years of age	0 (0)	5 (6)	67 (83)	9 (11)	
Individual practice	0 (0)	3 (12)	22 (88)	0 (0)	NS
Group practice	1 (1)	8 (8)	79 (82)	9 (9)	
Total (*n* = 122)	1 (1)	11 (9)	101 (83)	9 (7)	

^†^Fisher’s exact test.

*p < 0.05; NS: not significant.

When questioned about their level of knowledge concerning TPT work, 47% declared having very good knowledge and 53% had vague knowledge (Table 2).

Respondents (80.5%) correctly answered at least four questions. Most respondents correctly answered four out of six questions about TPT (74–99%) work (Figure 3). Only 48% of the GPs knew that the employer might refuse TPT work. No significant difference was observed according to age or practice type. Moreover, 13% of GPs knew that TPT work does not have to follow full-time sick leave. The percentage of correct answers for this question was higher among GPs younger than 45 years (22% vs 9%; *p* < 0.05), whereas it did not differ according to the type of practice. Physicians with a very good level of knowledge concerning TPT work tended to have a better total score than those with a vague level of knowledge (4.03/6 vs 4.23/6, NS).

Moreover, 90% of the GPs reported that they had frequently (83%) or regularly (7%) prescribed TPT work. The percentage of GPs who said that they prescribed periodically TPT work was significantly higher in the ≥45 years age group (11% vs 0%; *p* < 0.05) (Table 3). GPs with a better level of knowledge about TPT work also reported having a better level of use: All those GPs with a very good level of knowledge and 81% of the GPs with only a vague level of knowledge (*p* < 0.001) reported a ‘frequent’ or ‘regular’ level of use.

## Discussion

### Main findings

This study demonstrates that the level of knowledge and PRW consultation use and TPT work by GPs in a French department is good but is not optimal. Indeed, among those GPs interviewed, only 32% reported having a very good level of knowledge of PRW and 47% of TPT. Those with a very good level of knowledge reported a ‘regular’ level of use of PRW (51%) and TPT (7.4%) in the case of a difficult return to work. GPs who claimed to have a better level of knowledge of these two tools used them more frequently. Finally, we found that GPs did not systematically contact the OP.

### Strengths and limitations

Despite the absence of demographic data for the department, we believe that the sample population was comparable to that of GPs in the Pays de Loire region (of which Maine et Loire is one of five departments), both in terms of gender (34% women vs 39% in the region) and age (average age 50 years vs. 52)[11]. The characteristics of the regional population of GPs are comparable to the national GP population (36% female and average age 53 years)[12].

The response rate (61%) was high for a study on GP practices. This study was declarative, with the risk of not accurately reflecting reality. Notably, the notion of a ‘situation where returning to work appears complicated’ remained subjective. It is also difficult to directly transpose these results to other countries since health systems differ.

### Interpretation

It is possible to reflect on the role of the GP in the management of work-related health problems, which are relatively frequent particularly in common work-related psychiatric disorders, for which the prevalence has been reported, in a French study, to be 25% [13]. GPs are often the first health professional contacted by patients with work-related health problems. Still, they may encounter difficulties when prescribing an initial period of sick leave or encounter problems with return to work [14,15]. GPs, therefore, need to link the health problem to work, decide whether the sick leave is necessary, and if so, how long it should last, and then also consider the return to work [16].

In 2016, the European Agency for Safety and Health at Work classified the return-to-work systems of the OECD countries into four groups [17]. The Scandinavian countries form part of the first group having ‘a comprehensive and mature framework for rehabilitation and return to work, targeting all workers and valuing early intervention and individualised approaches’. These countries are characterised in particular by the development of a return-to-work plan at an early stage. Links with the employee’s company are therefore maintained throughout the sick leave period. These plans may include adjusting the employee’s workstation, working hours, or retraining within the company. Like United Kingdom (UK), France is in the second group, having a well-developed framework for rehabilitation and return to work, but ‘return-to-work considerations are generally only dealt with in a targeted manner at the end of the sickness absence, with limited provisions for early intervention’. The other two groups concern countries that focus on the care of people with disabilities.

In the UK, the ‘Fit Note’ changes the sickness certification process. GPs must determine whether the patient is ‘unfit’ or ‘maybe fit’ by specifying the conditions for a return to work [18,19]. More frequent and early use of the pre-return visit could enable France to approach the characteristics of the first group of countries, with early detection and establishing a return-to-work plan as recommended by the French guidelines.

Our results concerning PRW are consistent with those of the INPES study, in which 49% of interviewed GPs stated that they requested PRW in the case of prolonged sick leave [9]. Conversely, in the study by Tone et al., in another region of France in 2014, OPs reported that the PRW consultation was underused by GPs [10].

Our results on the frequency of contact between GPs and OPs are consistent with those of the INPES study, in which 71% GPs stated that they ‘often’ or ‘always’ suggest to their patients to contact the OP during recurrent periods of sick leave [9]. As the OP is at the centre of the French stay-at-work system, exchanges between GPs and OPs should be encouraged, which most GPs wish [9,20]. The relationship between OPs and GPs may be improved by facilitating the identification of the OP’s patients, GP’s perception and trust of OPs and establishing rules for exchange [9,21,22].

### Implications

The finding that GPs who claimed to have better knowledge used these tools most often suggests a need for increased training. In France, medical studies are divided into three cycles: a first theoretical cycle, a second cycle including hospital internships and the third cycle of specialisation with outpatient care for future GPs. Occupational health teaching, delivered in the second cycle, is not contextualised. During the third cycle, contextualised work placements and theoretical teaching vary depending on the particular faculty, and even within the same faculty, because it is often optional. However, it would be desirable that occupational health training be linked to real situations to adapt training to student needs better. It should be performed in cooperation with occupational health services and OPs to enhance understanding of their role and allow students to become familiar with interdisciplinary work. During continuing training, the traditional in-person training could also be enriched by sessions involving GPs and OPs and further complemented by diversified sources of information [23], each having different effects on their practices [24–27]. For example, a 3-h interactive programme was found to be followed by ‘a significant increase in GPs confidence in managing consultations on work and health’ [28]. In the UK, GPs trained in the Diploma in Occupational Medicine were found to more often use the ‘fit note’ [19]. However, the training of GPs should not be the only solution, as shown in a randomised trial in the Netherlands, where GPs play no formal role in the certification of sickness, which reported no benefit in terms of GPs registering work-related problems [29].

Lastly, another way to improve the use of these tools could be by implementating recording the use of PRW and TPT work by GPs, as is done for sick leave, to provide feedback to physicians and implement a policy of incentives.

## Conclusion

The level of knowledge and use of return-to-work tools by GPs can be improved. Studying the obstacles to their use will require complementary qualitative studies that focus on the GPs who currently use them infrequently.

## Supplementary Material

STROBE statement
