# Supplementary material for: Pre-return to work consultation and therapeutic part-time work: Cross-sectional study on level of knowledge and use by general practitioners in France
Source: Eur J Gen Pract. 2021 Jul 14;27(1):158–65. doi: 10.1080/13814788.2021.1948007 (PMC8281089; doi:10.1080/13814788.2021.1948007)
Supplement: STROBE statement [file IGEN_A_1948007_SM6489.docx]

STROBE Statement—checklist of items that should be included in reports of ***cross-sectional studies***

|  | Item No. | Recommendation | Page  No. | | Relevant text from manuscript | |
| --- | --- | --- | --- | --- | --- | --- |
| **Title and abstract** | 1 | (*a*) Indicate the study’s design with a commonly used term in the title or the abstract | 1 | | Title line 1 | |
|  |  | (*b*) Provide in the abstract an informative and balanced summary of what was done and what was found | 1 | | Abstract | |
| Introduction | | | |  | |  |
| Background/rationale | 2 | Explain the scientific background and rationale for the investigation being reported | 3 | | Lines 2-14 | |
| Objectives | 3 | State specific objectives, including any prespecified hypotheses | 4 | | Lines 14-15 | |
| Methods | | | |  | |  |
| Study design | 4 | Present key elements of study design early in the paper | 4 | | Lines 19-24 | |
| Setting | 5 | Describe the setting, locations, and relevant dates, including periods of recruitment, exposure, follow-up, and data collection | 5 | | Lines 2-6 | |
| Participants | 6 | (*a*)  Give the eligibility criteria, and the sources and methods of selection of participants | 4 | | Lines 19-24 | |
| Variables | 7 | Clearly define all outcomes, exposures, predictors, potential confounders, and effect modifiers. Give diagnostic criteria, if applicable | 5 | | Lines 9-17 | |
| Data sources/ measurement | 8* | For each variable of interest, give sources of data and details of methods of assessment (measurement). Describe comparability of assessment methods if there is more than one group | 5 | | *Lines 9-17* | |
| Bias | 9 | Describe any efforts to address potential sources of bias |  | |  | |
| Study size | 10 | Explain how the study size was arrived at | 4 | | Line 22-24 | |

Continued on next page

| Quantitative variables | 11 | Explain how quantitative variables were handled in the analyses. If applicable, describe which groupings were chosen and why |  |  |
| --- | --- | --- | --- | --- |
| Statistical methods | 12 | (*a*) Describe all statistical methods, including those used to control for confounding | 5 | Lines 20-22 |
|  |  | (*b*) Describe any methods used to examine subgroups and interactions |  | NA |
|  |  | (*c*) Explain how missing data were addressed |  | NA |
|  |  | (*d*) *Cohort study*—If applicable, explain how loss to follow-up was addressed  *Case-control study*—If applicable, explain how matching of cases and controls was addressed  *Cross-sectional study*—If applicable, describe analytical methods taking account of sampling strategy |  |  |
|  |  | (*e*) Describe any sensitivity analyses |  |  |
|  | | | | |
| Participants | 13* | (a) Report numbers of individuals at each stage of study—eg numbers potentially eligible, examined for eligibility, confirmed eligible, included in the study, completing follow-up, and analysed | 4 | Lines 21-24 |
|  |  | (b) Give reasons for non-participation at each stage |  | NA |
|  |  | (c) Consider use of a flow diagram | Figure 1 | Fig 1 |
| Descriptive data | 14* | (a) Give characteristics of study participants (eg demographic, clinical, social) and information on exposures and potential confounders | 4 | Line 19-21 |
|  |  | (b) Indicate number of participants with missing data for each variable of interest |  | No missing data |
| Outcome data | 15* | Report numbers of outcome events or summary measures | 6 | *Lines 5-24* |
|  | 16 | (*a*) Give unadjusted estimates and, if applicable, confounder-adjusted estimates and their precision (eg, 95% confidence interval). Make clear which confounders were adjusted for and why they were included | 6 |  |
|  |  | (*b*) Report category boundaries when continuous variables were categorized |  | NA |
|  |  | (*c*) If relevant, consider translating estimates of relative risk into absolute risk for a meaningful time period |  | NA |

Continued on next page

| Other analyses | 17 | Report other analyses done—eg analyses of subgroups and interactions, and sensitivity analyses | 6,7 | Line 19-24 and 10-14 |
| --- | --- | --- | --- | --- |
| Discussion | | | | |
| Key results | 18 | Summarise key results with reference to study objectives | 7 | Lines 18-23 |
| Limitations | 19 | Discuss limitations of the study, taking into account sources of potential bias or imprecision. Discuss both direction and magnitude of any potential bias | 8 | Lines 6-9 |
| Interpretation | 20 | Give a cautious overall interpretation of results considering objectives, limitations, multiplicity of analyses, results from similar studies, and other relevant evidence | 8-9 | Lines 11-24 and 1-16 |
| Generalisability | 21 | Discuss the generalisability (external validity) of the study results | 8 | Lines 8-9, 18-24 |
| Other information | |  | | |
| Funding | 22 | Give the source of funding and the role of the funders for the present study and, if applicable, for the original study on which the present article is based |  | Not applicable |

*Give information separately for cases and controls in case-control studies and, if applicable, for exposed and unexposed groups in cohort and cross-sectional studies.

**Note:** An Explanation and Elaboration article discusses each checklist item and gives methodological background and published examples of transparent reporting. The STROBE checklist is best used in conjunction with this article (freely available on the Web sites of PLoS Medicine at http://www.plosmedicine.org/, Annals of Internal Medicine at http://www.annals.org/, and Epidemiology at http://www.epidem.com/). Information on the STROBE Initiative is available at www.strobe-statement.org.
